# Adsorption of cationic dye onto Raphanus seeds: optimization, adsorption kinetics, thermodynamic studies

**DOI:** 10.1038/s41598-024-66761-z

**Published:** 2024-08-01

**Authors:** W. A. Hammad, M. A. Darweesh, Nasser Zouli, Samir M. Osman, Basant Eweida, M. H. A. Amr

**Affiliations:** 1 Faculty of Engineering, Tanta University, Tanta, Egypt; 2 Department of Chemical Engineering, Faculty of Engineering, Jazan University, Jazan, Saudi Arabia; 3 Pharmacognosy Department, Faculty of Pharmacy, October 6 University, Giza, Egypt; 4 Modeling and Simulation Research Department, Advanced Technology and New Materials Institute (ATNMRI), City of Scientific Research and Technological Applications, New Borg El-Arab, Egypt; 5 Tanta Higher Institute of Engineering and Technology, Tanta, Egypt

**Keywords:** Adsorption, Cationic dye, Methylene blue, Raphanus seeds, Contamination, Wastewater treatment, Adsorption isotherm, Adsorption kinetics, Pollution remediation, Chemistry, Engineering

## Abstract

Dyes are one of the common contaminants in industrial wastewater. Adsorption is the most widely method which used to treat dye-contaminated water due to their easy use, cost-effectiveness, and their efficiency was high. The aim of this study is the investigating of the utilization of the activated carbon which prepared from Raphanus seeds solid residual (ACRS) as a low cost adsorbent for removing of cationic Methylene Blue dye (MB)from wastewater. measuring the surface area using BET methods and SEM. The FT‒IR and XRD was measured. Different variables (e.g.: initial concentration of the dye, pH, contact time, and dosage) have been studied. Process has been systematically investigated experimentally at (25 ± 1 °C). The % removal of MB reached 99.4% after 90-min MB adsorption (40 mg/L) was observed within 5 min of contact time for the Raphanus seeds solid residual (ACRS) dosage of 4 g/L. MB initial concentration (10 ppm) Raphanus seeds solid residual (ACRS) effectively adsorbed MB (> 99%) over a widely range of pH (from pH 2 to pH 8). However, a swift decline in removal was observed when the pH was set at 7. The results of the adsorption kinetics analysis indicate a strong correlation with the pseudo-second-order model, as evidenced by the high regression coefficients. However, the adsorption capacity diminished with a rise in temperature. Thermodynamic calculations of (MB) onto Raphanus seeds solid residual (ACRS) is an exothermic reaction. The results have been indicated that the effectiveness of MB removal by activated carbon prepared from Raphanus seeds solid residual is favorable under neutral conditions, Raphanus seeds solid residual (ACRS) can be considered an efficient, environmentally friendly, readily available, and economical adsorbent that could treat industrial wastewater contaminated with cationic textile dyes. The objective of the experiments was to investigate the impact of various factors on the response of a process or formulation. To accomplish this goal, response surface methodology (RSM) has employed as a statistical model. RSM is an efficient and effective method for optimizing processes through the use of a quadratic polynomial model. The utilization of RSM allows for a reduction in the number of experiments needed, thus minimizing the associated costs of extensive analysis. This method has been done using Box–Behnken Design (BBD) to optimize % removal of MB. The optimal conditions as obtained from the RSM is pH 7,contact time  120 min, initial concentration  10 ppm, ACRS dosage  1 g, adsorption temperature  45 °C.

## Introduction

The mention of the need for effective methods to remove dyes from wastewater in line 7 is intricately linked to the broader context of dye contamination discussed in the preceding sentences. In recent times, water pollution has emerged from various sources, both natural and anthropogenic, including industrial discharges, agricultural runoff, sewage discharges, and oil spills. The comprehensive management of this issue necessitates a multifaceted approach, involving strategies such as wastewater treatment, the enforcement of regulations and policies, and public awareness and education programs^1^.

The persistence of pollutants, highlighted in the context of dye contamination, emphasizes their enduring presence in the environment and the potential for bioaccumulation in organisms. This persistence underscores the urgency for the development and implementation of efficient methods to remove dyes from wastewater. The significance of this endeavor is underscored by its role in safeguarding aquatic life and preserving the overall environmental integrity. Various approaches, spanning physical, chemical, and biological methods, exist for the removal of dyes. However, achieving an optimal and sustainable solution requires a comprehensive understanding of dye removal mechanisms, careful consideration of potential trade-offs, and an assessment of economic, social, and environmental factors^2^. This holistic approach ensures that the chosen methods not only effectively remove dyes but also align with broader goals of sustainability and environmental protection.

Furthermore, the release of these dyes into the environment carries health risks for humans. Contaminating drinking water sources and adversely affecting crops and livestock that consume the contaminated water, the presence of these dyes poses a direct threat to public health. Hence, the call for effective methods to remove dyes from wastewater is intricately connected to the broader narrative of addressing water pollution and safeguarding both the environment and human well-being.

The large-scale production of textiles and clothing leads to significant amounts of dye-containing wastewater being generated and released into the environment, potentially contaminating local water sources and having adverse impacts on the surrounding ecosystems and human health. To mitigate these negative effects, various treatment methods developed as physical, chemical, and biological methods^3–5^.

MB is cationic dye which has used in textile industry, but it is harmful to the environment and human health. It has been linked to toxicity, mutagenicity, and potential cancer-causing effects. Therefore, it is important for removing MB from textile effluent in order to create a cleaner and safer environment^6^.

**Figure 1:**
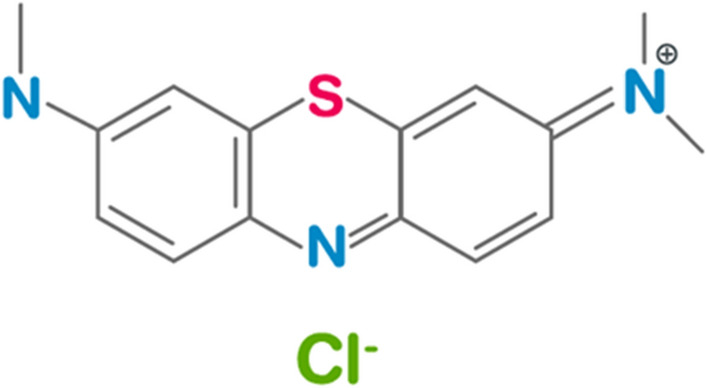
Structure of MB.

MB has been chosen in this study because it is an adsorbent with strong adsorption onto solids. Its molecular weight is 373.9. The structure of MB was given below^7^ in Fig. 1.

**Figure 2:**
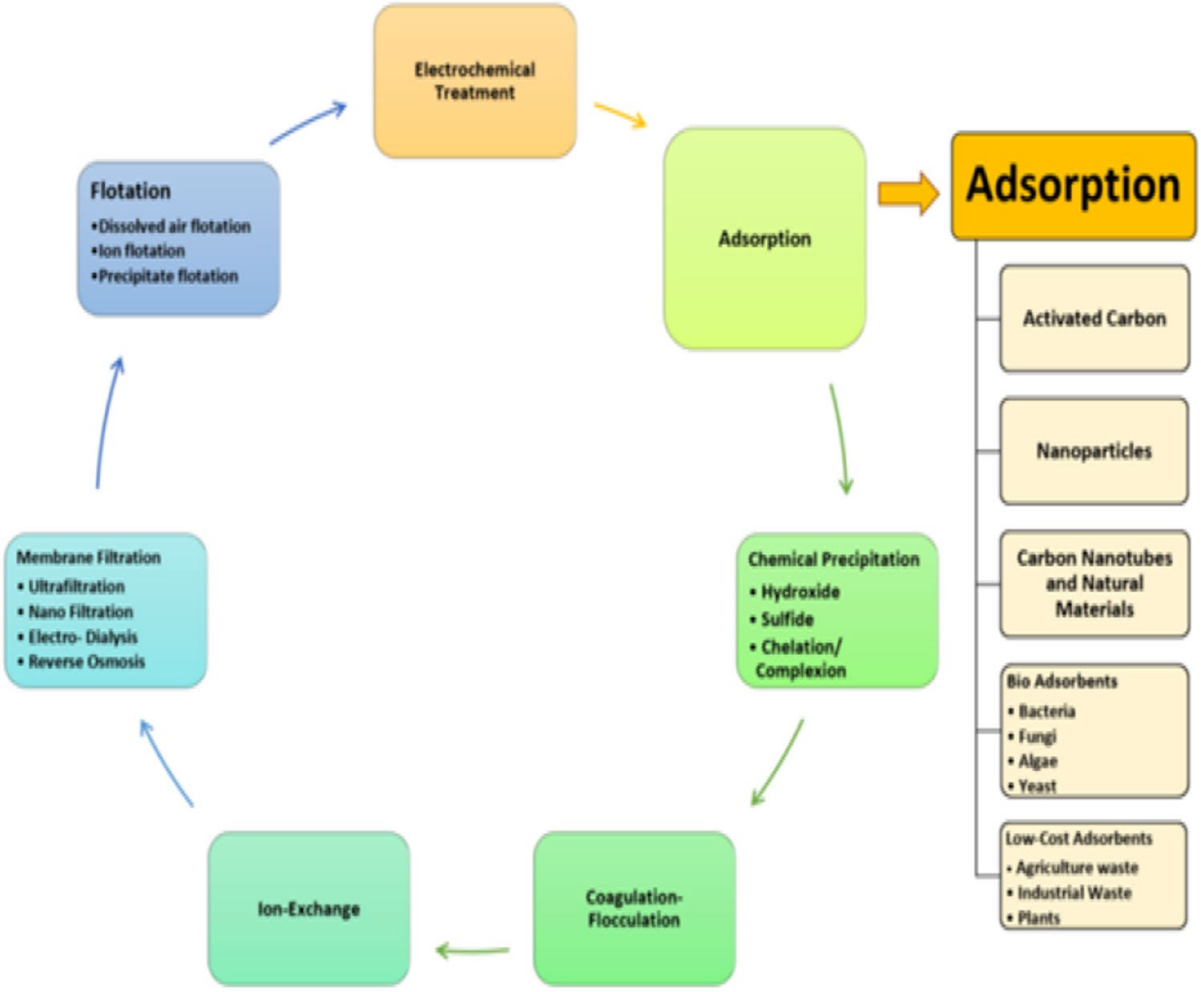
Tertiary treatment schematic diagram for wastewater treatment.

Multiples of chemical-physical methods which are the flocculation, the coagulation, the membrane, the filtration and the adsorption processes are available and used for the treatment of the textile wastewater. Figure 2 shows the different methods for wastewater treatment.

The existing research on the development of low-cost adsorbents is not thoroughly expounded upon. It is imperative to delve into this area, considering its significance in the context of wastewater treatment. Adsorption stands out as one of the most economical and effective methods for dye removal from wastewater. The choice of adsorbent material, whether natural (e.g., activated carbon) or synthetic (e.g., zeolites, clay minerals, or metal oxides), depends on specific application needs, considering factors like pollutant type and concentration, as well as the desired level of decontamination^7^^.^

The efficacy of the adsorption method, particularly utilizing activated carbon, is widely acknowledged due to its extensive surface area, impressive adsorption capacity, and varied pore size distribution. However, the high cost associated with commercial activated carbon poses a constraint on its widespread utilization. Consequently, there is an urgent demand for exploring low-cost alternative adsorbents that can efficiently remove dyes from wastewater. The ongoing research in this field is focusing on the development of low-cost alternative adsorbents derived from agricultural wastes, industrial waste, and by-products, garnering increased attention in recent years^8,9^. This research direction not only addresses the economic challenges associated with conventional adsorbents but also aligns with sustainability goals by repurposing waste materials for environmental benefit.

**Figure 3:**
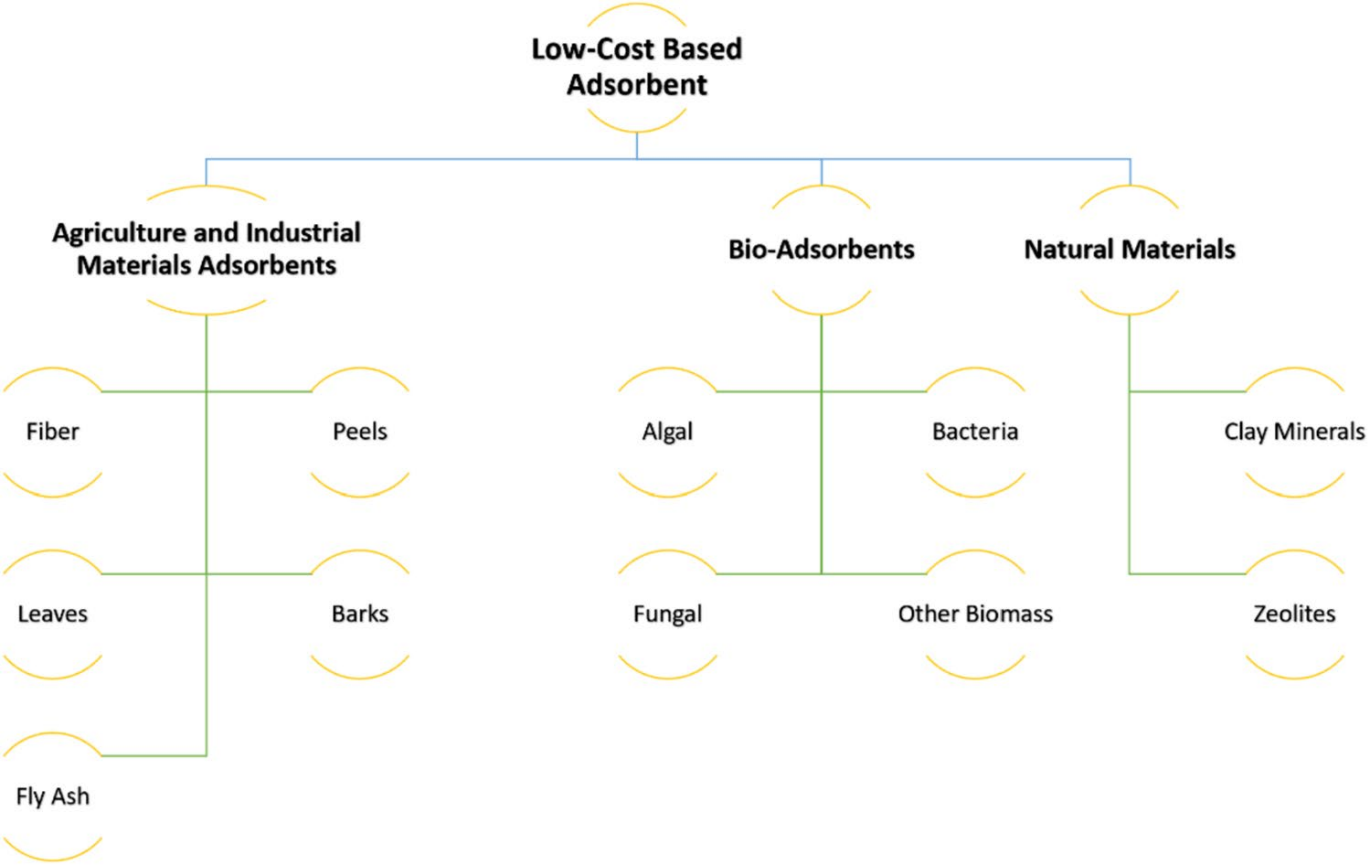
Schematic diagram of low-cost adsorbents can be used in wastewater treatment.

**Figure 4:**
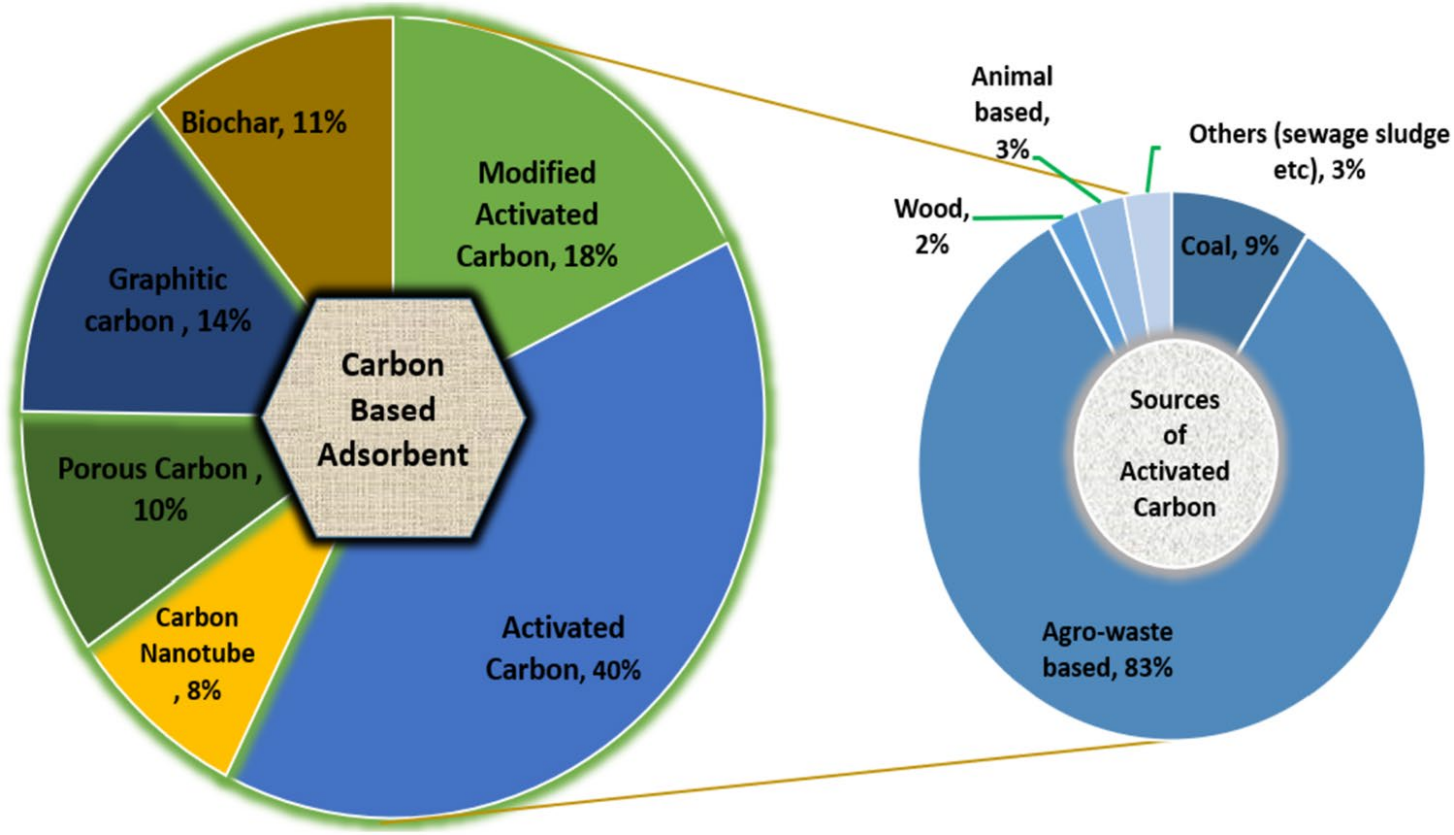
Sources of activated carbon for MB elimination^10^.

Figure 3 illustrates some low-cost adsorbent^10^. Activated carbon stands out as a potent adsorbent material for removing dyes from wastewater.^9^ Fig. 4 shows the carbon used as adsorption material in MB removal from 2008 to 2021^10^^.^

The industrial processes of squeezing oils produce residuals from the squeezing process in large quantities cause serious environmental damage. The use of these residuals from oil extraction as a sorbent for removing the dyes from wastewater of the textile industry provides a solution to both the disposal of the residuals and the removing dyes from the environment. This process can be done through adsorption, where the residuals act as a sponge to absorb the dyes from the wastewater. The use of these residuals not only provides a solution to environmental problems but also adds economic value to what was previously considered waste.^9^.

The aim of this study is the investigating of the utilization of the activated carbon which prepared from Raphanus seeds solid residual (ACRS) as a low cost adsorbent for removing of cationic methylene blue dye (MB) from wastewater.

## Experimental study

### Materials

The chemicals utilized in this investigation procured from the Research-Lab of Fine Chemical Industries. Preparation of solutions was conducted depending on established protocols, and distilled water was utilized throughout the experiment.

### Adsorbent material preparation

Raphanus seeds were washed and then dried in the oven at 110 °C to eliminate any moisture content. The squeezing process for the seeds was executed to extract any oil content. The solid residual from this process underwent a drying process in the oven for 5 h at 100 °C and was subsequently incinerated in a muffle furnace at 400 °C for 3 h.

### MB stock solution preparation

A 1000 mg/L stock solution of methylene blue (MB) was prepared by dissolving 1 g of MB in 1 l of distilled water. Dilutions of the MB stock solution were achieved by adding distilled water following the principles of dilution. The pH of the solutions was adjusted to the desired values by the addition of (0.1 N) NaOH or 0.1 N H_2_SO_4_, maintaining a constant temperature of 25 °C.

### Adsorption experiment

**Figure 5:**
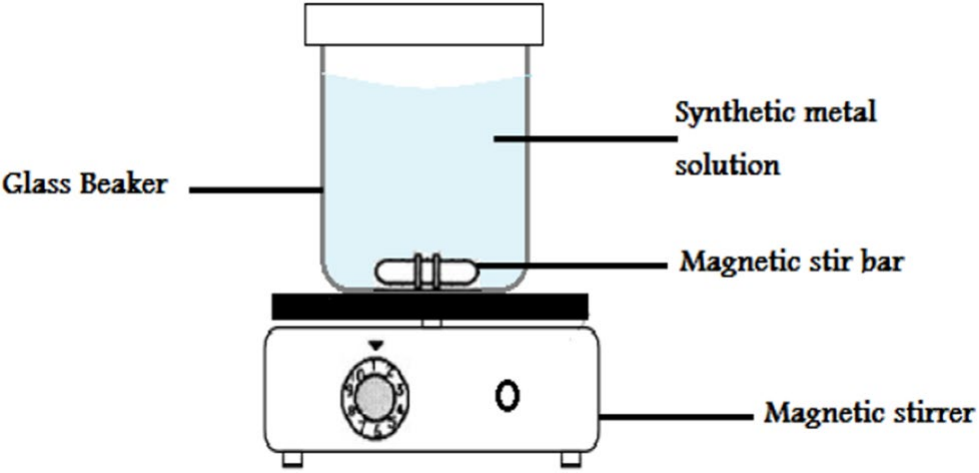
A schematic drawing of the device which has been applied to the adsorption process.

The experiments were conducted in batches, stirring Raphanus seeds solid residual (ACRS) from Raphanus seeds with a digital magnetic stirrer MS-H-Pro using a Teflon bar (2 cm length) with a temperature sensor PT 1000 and 250 ml of an aqueous solution of MB at a stirring speed of 300 rpm. Varying pH values from 2 to 8 were employed to understand the pH impact on MB adsorption. The experiments were executed at different temperatures (25, 30, 35, 40, and 45 °C), various initial concentrations (10, 20, 30, 40, and 50 ppm) for MB, and different weights of Raphanus seeds solid residual (ACRS) = (0.1, 0.3, 0.5, 0.7, and 1 g/250 ml). Five milliliters of aliquots were taken from the reaction solution at different time intervals for MB analysis over a contact time of 90 min. The samples were taken at regular time intervals. The UV–Visible spectrophotometer (Varian Flame AA240 Fs; Graphite GTA 120) was used to record the MB concentration at the wavelength of 664 nm. Figure 5 depicts the results.

The % Removal (% adsorption) has been calculated using Eq. (1)

1 $$\boldsymbol{\%} Removal=\text{\% adsorption}=\left(\frac{{C}_{0}-{C}_{t}}{{C}_{0}}\right)\times 100$$

C_o and_ C_t_ are the initial concentration and the concentration at the equilibrium of MB solution at (t) time (ppm) respectively.

The adsorption parameters consist pH, time(min), concentration of MB (ppm), and Dose (mg/250 ml). The response surface approach i.e. (Box-Behnken's design) has been used for optimization of % removal percent of MB using activated carbon from Raphanus seeds residual^11^.

### Box–Behnken design

Box–Behnken design with four factors were examined at three levels (−1, 0 and 1)^12,13^. Table 1 illustrates the elements and the levels, taking into four different variables, using the Box–Behnken design of the experiments (BBD**),** For the statistical analysis, there were a certain number of experiments needed (twenty-seven experimental runs which were carried out to predict the result of five independent factors pH, A (2–7), time(min), B (0–90), concentration of MB (ppm),C (10–50), and Dose (mg/100 ml), D (0.1–0.5) on % removal of the MB from in solution. Experimental conditions and the results are shown in Table 1. A regression analysis was performed to generate a model for the response, and its efficacy was evaluated through the use of ANOVA and F-tests^14^. the evaluation and improvement of the data process were carried out utilizing the Statistica software, and the interaction effects were represented through the application of a quadratic equation (Eq. 2)

2 $${\text{Y}} = \beta_{0} + \sum \beta_{{\text{i}}} {\text{x}}_{{\text{i}}} + \, \sum \, \beta_{{{\text{ii}}}} {\text{x}}_{{\text{i}}}^{{2}} + \, \sum \beta_{{{\text{ij}}}} {\text{x}}_{{\text{i}}} {\text{x}}_{{\text{j}}}$$

**Table 1:** Box–Behnken factorial design levels for experimental variables.

Variable code	Variables	Levels		
		− 1	0	+ 1
A	pH	2	5	7
B	Time (min)	0	30	90
C	Concentration of MB (ppm)	10	30	50
D	Dose (mg/250 ml)	0.1	0.5	1

The variables in the equation are defined as follows: Y represents the predicted response, β0 is the intercept term, βi represents the linear effect, βii represents the square effect, and βij represents the interaction effect.

### Surface study

The sample's surface area was calculated from N_2_ adsorption which measured at -196 oC using the BELSORP equipment, Japan. The sample was initially out-gassed under vacuum (10^–4^ Torr) at 300 °C. The BET surface area (S_BET_) was calculated by the BET equation^15^.

The samples' surface morphology was analyzed using a Scanning Electron Microscope (SEM) model (Quanta 250 FEG, manufactured by FEI Company in the Netherlands). The instrument was employed at an acceleration voltage of 30 kV to increase the resolution of the images. To reduce sample charging due to the electron beam, a 3.5 nm layer of gold was deposited onto the sample surface, effectively minimizing the charging effects.

### The FT‒IR and XRD measurement 

The Fourier transform infrared (FT-IR) spectrum of the sample was obtained using a PerkinElmer Spectrum 2 instrument. Additionally, the powder X-ray diffraction (XRD) analysis of the pattern was conducted using a PW3040/60 P analytical diffractometer.

## Results and discussion

### The effect of pH

**Figure 6:**
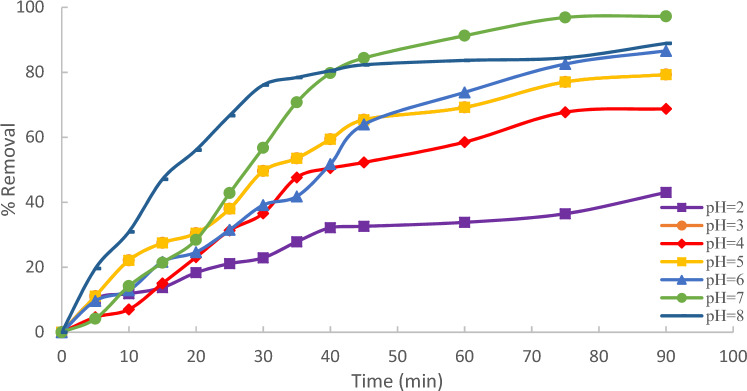
pH effect regarding MB adsorption onto Raphanus seeds solid residual (ACRS) (initial concentration = 10 ppm, Raphanus seeds solid residual (ACRS) dose = 0.5 g/250 ml, stirring speed = 300 rpm T = 25 ℃ and time = 90 min).

Evaluate the pH at the point of zero charge (pzc) for the utilized adsorbent. The pH of the solution plays a crucial role in the adsorption process, particularly in the extraction of methylene blue (MB) from an aqueous solution. It influences surface charges, ionization degree, and Adsorbate specification of the adsorbent. Initial parameters for MB adsorption, including a concentration of 10 ppm and a contact time of 90 min, were established to achieve equilibrium. Equilibrium studies, conducted at varying pH values from 2 to 8, aimed to comprehend the pH impact on MB adsorption. The pH of solutions was measured using the HANNA pH 211-Romania pH-meter which clear in Fig. 6.

**Figure 7:**
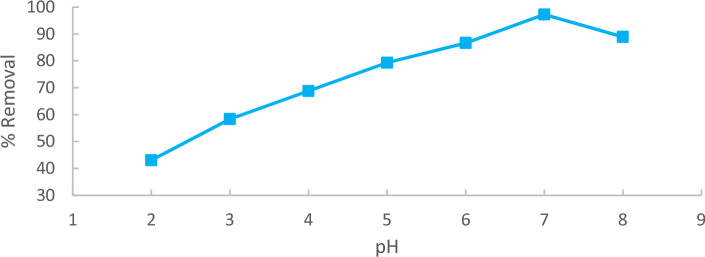
pH effect regarding MB adsorption.

The observed increase in pH correlated with a higher % removal^16^), attributed to the acidic state of the bioadsorbent. As the pH increases, the adsorbent's surface becomes negatively charged, leading to an increased adsorption capacity^17^, reaching its peak at pH  7. Results demonstrate a significant rise in the percentage removal of methylene blue (MB) from 82.7% to 99.4% as the solution's pH increases from 2 to 7. Beyond pH 7, the % removal of MB decreases with an increase in the solution's pH, indicating a reduction in adsorption capacity. The maximum removal obtained at pH  7 was 99.4%, as shown in Fig. 7.

### Effect of initial concentration

**Figure 8:**
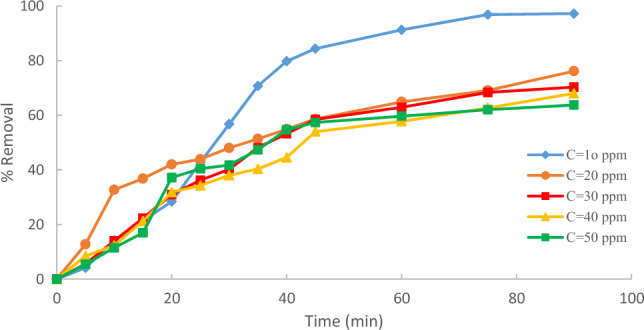
Effect of % removal of Raphanus seeds solid residual (ACRS) (pH  7, Raphanus seeds solid residual (ACRS) with time at different MB initial concentration dose = 0.5 g/250 ml, stirring speed = 300 rpm T = 25 ℃ and time = 90 min).

An investigation into the effect of initial methylene blue MB concentration on its removal onto Raphanus seeds solid residual (ACRS) was performed under controlled conditions. The conditions were maintained at a temperature of 25 °C, a pH of 7, a contact time of 90 min, and an agitation speed of 300 rpm. adsorbent dose = 0.5 g/250 ml for different MB initial concentrations (10,20,30,40and 50 ppm), As depicted in Fig. 8, the percentage removal of methylene blue (MB) decreases with an increase in the initial MB concentration. At low concentrations, MB is adsorbed on the vacant sites of the adsorbent surface and these places are saturated and filled by increasing the concentration. When methylene blue's initial concentration is low, an abundance of active spots on the adsorbent's surface become available for dye adsorption. However, if the initial concentration of MB dye is increased, the number of moles of the MB dye is higher than the number of vacant sites. Therefore, the available sites are quickly saturated and the dye removal rate decreases^4^.

**Figure 9:**
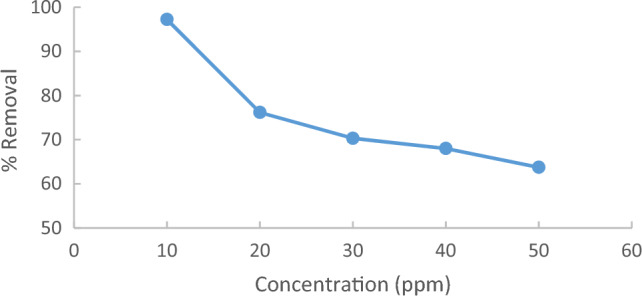
Effect MB initial concentration on adsorption.

The result reveals that as the initial concentration of methylene blue (MB) increased from 10 to 50 ppm, the percentage removal of MB decreased from 99.4 to 50.9%, as demonstrated in Fig. 9^18^.

### The effect of the adsorbent dose

**Figure 10:**
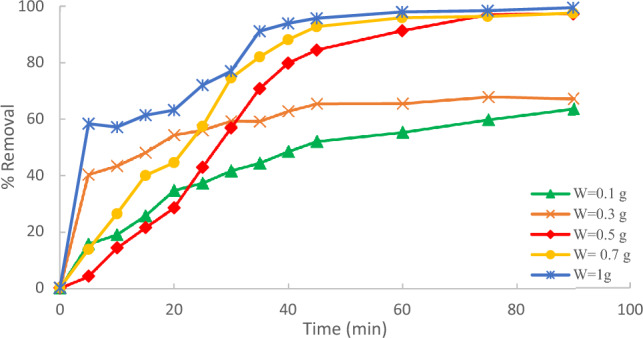
Effect of the adsorbent dose of Raphanus seeds solid residual (ACRS) on the % removal MB.

The impact of the adsorbent dose on the removal of methylene blue (MB) was evaluated at an initial MB concentration of 10 ppm, 90 min contact, and temperature of 25 °C. Different adsorbent doses (ACRS = 0.1, 0.3, 0.5, 0.7, and 1 g/250 ml). According to Fig. 10, the percentage removal of methylene blue (MB) increases with an increase in the amount of adsorbent used. This result is expected because a rise in the amount of adsorbent results in increased surface area with available adsorptive sites. at a fixed initial concentration of sorbate i.e. the increased accessibility increases the number of exchangeable sites or the surface area insured which enhanced the uptake of MB^18–20^.

**Figure 11:**
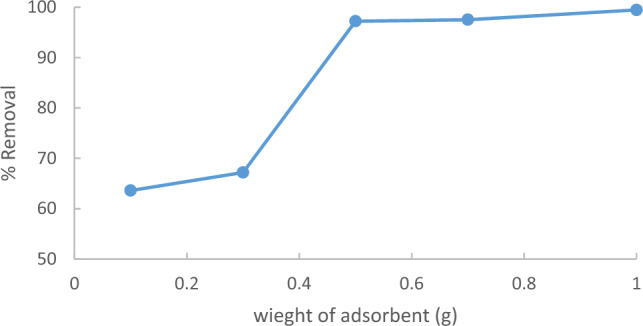
Effect of adsorbent dose % removal Methylene Blue.

The outcomes revealed that the uptake or percentage removal of methylene blue (MB) increased from 63.6 to 99.4% as the adsorbent dose increased from 0.1 g/250 ml to 1 g/250 ml, as illustrated in Fig. 11

### The effect of temperature

**Figure 12:**
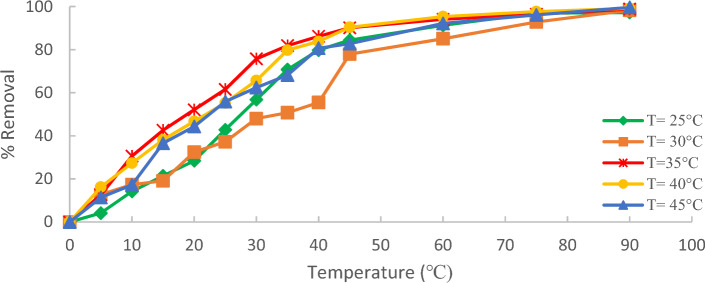
Effect of temperature on % removal of MB on (ACRS), initial MB concentration 10 ppm, adsorbent dose: 1 g/250 ml stirring speed 300 rpm, contact time 90 min and pH  7.

Figure 12 demonstrates the impact of temperature on the percentage removal of methylene blue (MB) from wastewater using (ACRS). Different temperatures (25, 30, 35, 40, and 45 °C) with an initial concentration of 10 ppm of MB were used in this study in the presence of (1 g/250 ml) for (ACRS). The figure indicated that within 90 min, the removal efficiency increased as the temperature increased because Higher temperatures facilitated elimination of methylene blue (MB) by promoting the adsorption at the coordination sites of the adsorbent. This was due to the speeding up of some previously slow steps and the creation of additional activation sites on the adsorbent surface^21–23^.

**Figure 13:**
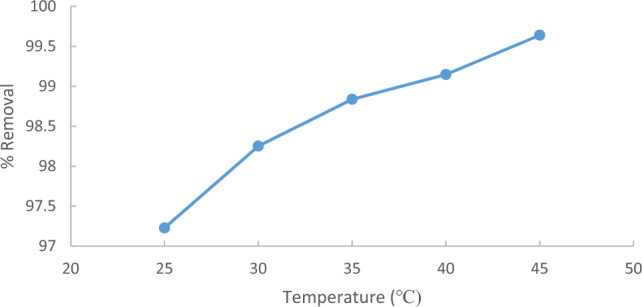
The effect of temperature on % the removal of the MB.

The result reveals that the uptake of a Fig. 13 demonstrates that the percentage removal of methylene blue (MB) increased from 97.22 to 99.64% with increasing the temperature from 25 to 45 °C.

### RSM analysis for optimization of % removal of the methylene blue from aqueous solution

The study of the removal of methylene blue was performed using a Box–Behnken factorial design-based response surface methodology (BBD). The initial step involved investigating the influence of individual variables through single parameter experiments. The levels of selected parameters were then used in a series of experiments, the results of which were recorded and analyzed using statistical plans and response procedures. The details of the selected the parameters' levels are presented in Table 1, while the results of the statistical analysis are outlined in Table 2

**Table 2:** Evaluation of operational factors in the Box–Behnken factorial design for Methylene Blue removal for optimization of Methylene Blue removal percent (%) using activated carbon from Raphanus seeds residual.

Trail	pH	Time(min)	Concentration of MB(ppm)	Adsorbent dose (gm/250 ml)	Removal %	
					Measured	Predicted
1	7	0	30	0.5	0.00	2.4983
2	7	30	10	0.5	56.76	52.3838
3	2	0	30	0.5	0.00	0
4	5	90	30	1.0	54.32	62.1428
5	7	30	50	0.5	41.74	34.1558
6	5	0	30	1.0	0.00	0
7	5	90	50	0.5	45.74	38.6819
8	5	30	30	0.5	22.22	22.2089
9	5	0	50	0.5	0.00	4.5854
10	2	30	50	0.5	7.90	10.7281
11	2	30	10	0.5	22.90	27.8761
12	5	30	30	0.5	22.22	22.2089
13	2	30	30	0.1	0.00	0.0426
14	5	90	10	0.5	79.29	76.1579
15	5	90	30	0.1	20.32	23.2753
16	5	30	50	1.0	26.35	25.5948
17	5	30	30	0.5	22.22	22.2089
18	2	30	30	1.0	6.41	5.4185
19	5	30	10	1.0	58.91	50.5348
20	5	30	50	0.1	2.10	9.83749
21	7	30	30	0.1	6.21	11.5578
22	5	0	10	0.5	0.00	0
23	2	90	30	0.5	16.32	13.9351
24	7	30	30	1.0	42.21	44.952
25	5	30	10	0.1	23.71	21.9183
26	7	90	30	0.5	70.32	71.5328
27	5	0	30	0.1	0.00	0

The results demonstrated that the % of removal of methylene blue in varying degrees of the four variables were different, due to the impact of various variable levels on the % of removal of MB. As indicated by the results in Table 2, the highest MB removal rate was achieved in test experiment 14, reaching a value of 79%. Additionally, the small discrepancy between the actual and predicted MB removal percentages reflects the high model accuracies^24,25^. As a result, the study employed a quadratic model of analysis of polynomial equations and the second order to establish the connection between the key elements and the response variable, which is the percentage removal of Methylene Blue, as the optimal model. The validation of the model was performed using ANOVA analysis and the results of variance analysis are presented in Table 3. Multivariate Regression Analysis of the experimental results resulted following second-order polynomial equation, represented as Equation (Eq. 3). The equation gotten through analysis of the experiment's outcomes using multiple regression (Eq. 3) is a second-order polynomial equation that can explain the % removal of Methylene Blue for the significant variables. The linear parameters in the equation are represented by the variables A, B, C, and D.; second-order parameters of the model were A^2^, B^2^, C^2^, and D^2^, along with the interaction parameters AB, AC, AD, BC, BD, and CD:

3 $${\text{Y}} = { 16}.0{736} - {34}.{\text{313A}} + 0.{\text{4519B}} - {1}.{\text{5233C}} + {31}.{\text{6555D}} + 0.{\text{2123A}}^{{2}} - 0.00{\text{42B}}^{{2}} + 0.0{\text{255C}}^{{2}} - {33}.{35}0{\text{8D}}^{{2}} + 0.{\text{1121AB}} - 0.00{\text{54AC}} + {6}.{\text{2263AD}} - 0.00{\text{82BC}} + 0.{3}0{\text{89BD}} - 0.{\text{3572CD}}$$

where the response variable is represented by "Y", which is the amount of Methylene Blue removed, and "A", "B", "C", and "D" are coding values for the significant test variables, including pH, time (min), the concentration of Methylene Blue (in parts per million), and the adsorbent dose (in grams per 250 ml), respectively.

**Table 3:** ANOVA results for % removal of Methylene Blue.

Source	Sum of square	df	Mean of square	F-value	p-value	Significant or insignificant model terms
Model	11,213.58	4	2803.396	16.21027	0.000003	Significant
A	2998.98	1	2998.982	43.21887	0.000026	Significant
B	5781.41	1	5781.410	83.31696	0.000001	Significant
C	1346.14	1	1346.142	19.39950	0.000859	Significant
D	1396.47	1	1396.472	20.12482	0.000745	Significant
A^2^	8.50	1	8.502	0.12252	0.732382	–
B^2^	288.16	1	288.156	4.15267	0.064238	–
C^2^	555.63	1	555.627	8.00724	0.015184	Significant
D^2^	235.96	1	235.956	3.40040	0.090000	–
AB	700.96	1	700.964	10.10172	0.007946	Significant
AC	0.30	1	0.301	0.00434	0.948556	–
AD	203.02	1	203.019	2.92575	0.112881	–
BC	235.03	1	235.025	3.38699	0.090563	–
BD	169.40	1	169.400	2.44125	0.144157	–
CD	41.67	1	41.675	0.60058	0.453358	–
Error	832.69	12	69.391	–	–	–
Total SS	15,018.25	26	–	–	–	–

The importance of the model was assessed using the F-value and p-value parameters. a significant F-value and a small p-value (less than 0.05) suggest that the model accurately predicts the experimental results. In this case, the F-value was 16.21027 and the p-value was 0.000003, indicating a completely significant model. Table 3 revealed that pH, contact time (minutes), initial methylene blue concentration (ppm), and adsorbent dose (g/250 ml) were significant factors affecting the % removal of methylene blue, with initial concentration being the most effective factor.

**Figure 14:**
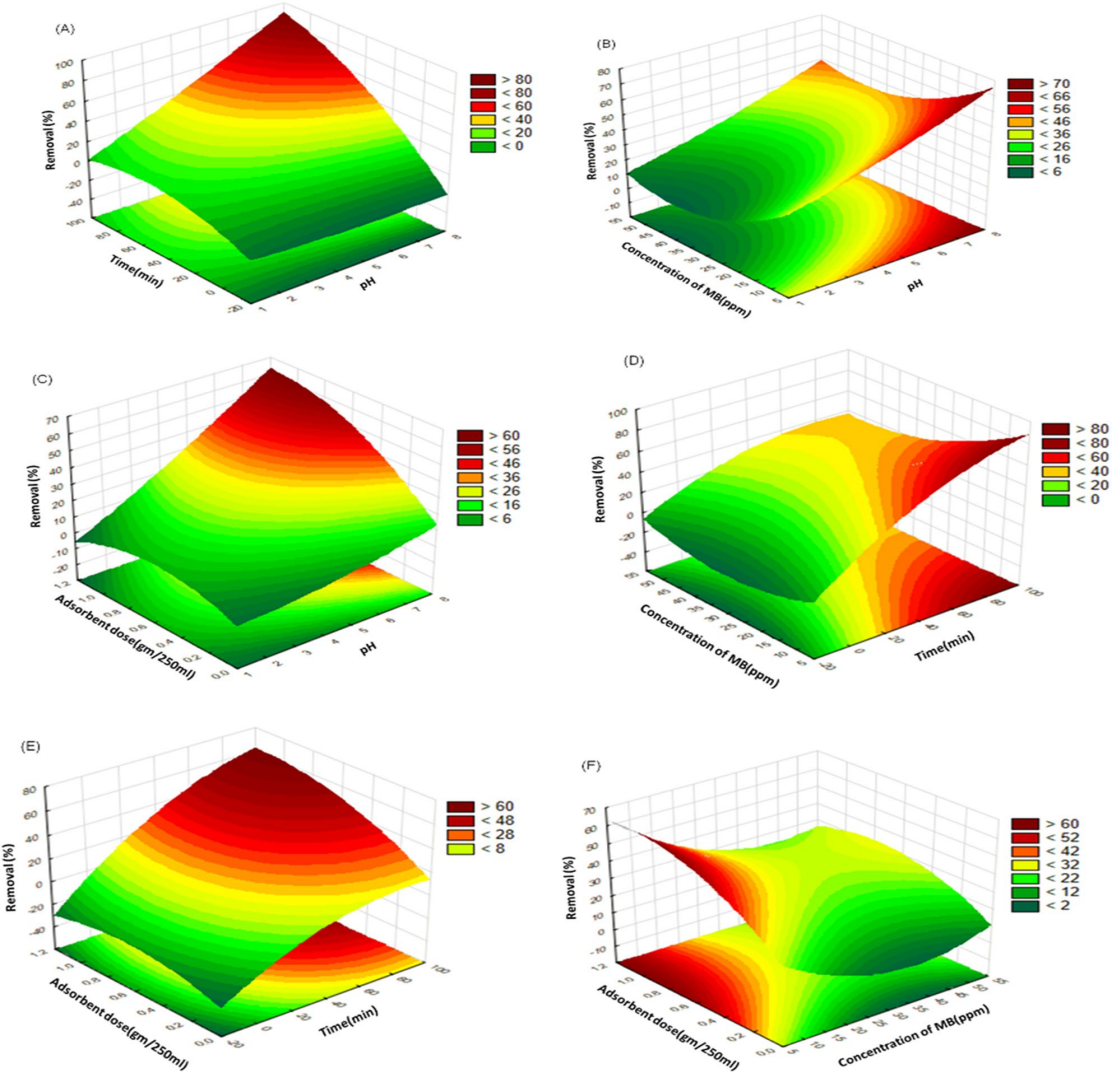
Response surface methodology of % removal of MB using activated carbon from Raphanus seeds residual: (**A**) time (min)/pH, (**B**) effect of concentration of MB (ppm)/pH, (**C**) adsorbent dose (g/250 ml)/ pH, (**D**) effect of concentration of MB (ppm)/time(min), (**E**) adsorbent dose (g/250 ml)/time (min), (**F**) adsorbent dose (g/250 ml)/concentration of MB (ppm).

The predictor variables have been plotted between the X and Y axes, with the response variable displayed on the Z axis, in order to identify the important impact of interactions among the various predictors (Fig. 14A–F) on % removal of MB using activated carbon from Raphanus seeds residual. The response surface's 2D contour plot have been generated in three dimensions for the pairwise combinations of the four factors (AB, AC, AD, BC, BD, CD), with the third factor kept at its central point level (0). As illustrated in Fig. 2E, the interactive effect of the dose and concentration resulted in the highest response.

Figure 14A, D, E demonstrates some variables (effect of contact time, pH, Conc. of MB, and Adsorbent dose on % removal of MB using activated carbon from Raphanus seeds residual which increased with lengthening the time from 0 to 90 min due to increasing the time, networks of the adsorbent where fast spread at the early stage and then reach to equilibrium after 90 min. So, it was much easier for MB molecules at the first time to penetrate inside the activated carbon from Raphanus seeds residual and include the adsorption sites showed an initial increase with time, however, with further progression, the adsorbent's surface-active groups began to decrease and the percentage removal of MB stabilized^26^.

### Characterization

The raw seeds, known as the residual solid from Raphanus seeds (ACRS), serve as a crucial component in the study. The characterization of these raw seeds is imperative for understanding their properties and potential applications.

The physical and chemical properties of ACRS were analyzed to provide comprehensive insights into its composition. Commonly assessed characteristics include morphology, particle size, surface area, elemental composition, and structural features. Techniques such as scanning electron microscopy (SEM), X-ray diffraction (XRD), Fourier-transform infrared spectroscopy (FTIR), and elemental analysis are commonly employed for characterization. Morphological studies using SEM reveal the surface topography, size, and shape of the ACRS particles. XRD provides information about the crystalline structure, while FTIR helps identify functional groups present in the material. Elemental analysis assists in determining the elemental composition, providing data on the presence of key elements.

This characterization is essential for establishing a baseline understanding of the raw seeds' properties, which, in turn, contributes to the interpretation of the adsorption process and the effectiveness of ACRS as an adsorbent.

#### Surface study

**Figure 15:**
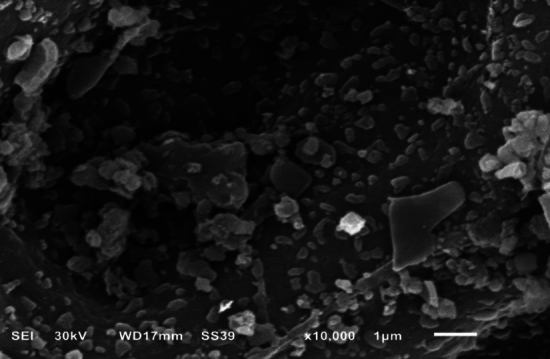
Scanning electron micrograph (SEM).

The surface area calculated by BET equation of the sample was 9.25 m^2^/g. The surface morphology studied by SEM (Fig. 15) shows that the sample is nonporous. It obvious, the structure of the sample has a small surface area. This is attributed to the simple and inexpensive preparation method that matches the environmental conditions, which is the direct burning of the sample at 400 degrees. The observed surface area is likely due to the small voids formed by the aggregation of small pieces of solid matter.

#### X-ray diffraction analysis (XRD)

**Figure 16:**
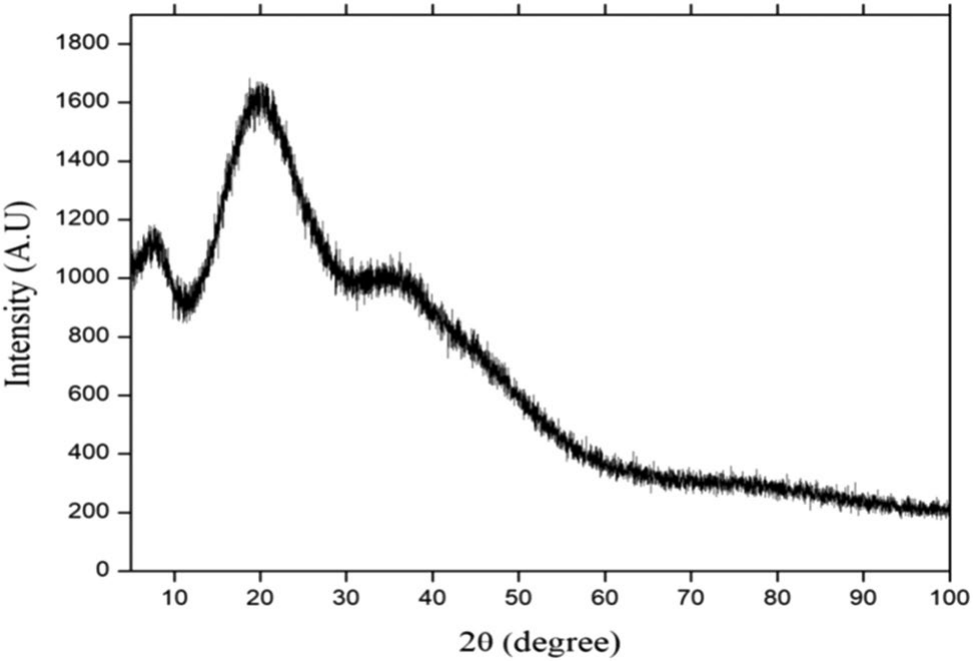
XRD pattern of Raphanus seeds residual powder.

Figure 16 presents the X-ray diffraction (XRD) pattern obtained from the radish seeds residual sample, which was exposed to Cu Kα radiation (λ = 1.54 Å) with a 2θ scanning range of 5 to 80 degrees. The broad peak observed at 2θ = 21.10 suggests that the radish seeds residual powder is amorphous in nature^27^. Additionally, the XRD pattern reveals that the crystalline structure of the radish seeds residual sample is disordered.

#### Fourier transform infrared spectra

**Figure 17:**
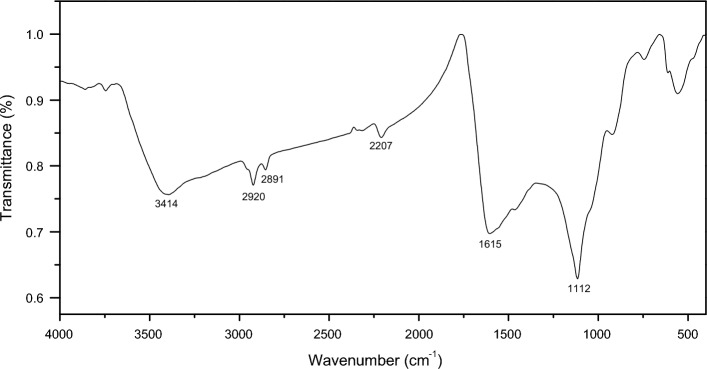
FT‒IR spectra of natural Raphanus seeds residual.

The FTIR spectrum of the sample has been recorded between 4000 and 400 cm^−1^ at resolution (0.1 cm^−1^)^28^. Figure 17 displays the FT-IR spectra of the natural radish seeds residual, with the vibrational assignments presented in Table 1. The broad band with intense intensity at 3414 cm^−1^ is a result of the hydroxyl (OH) group's stretching vibrations. The bands for the aliphatic C–H stretching occur between 2851 and 2920 cm^−1^. The stretching of the alkyne group is observed at 2207 cm^−1^, and the stretching of the carbonyl group is seen at 1615 cm^−1^. The stretching mode of the C=O group can be found at 1112 cm^−1^. FTIR spectral data indicate the presence of flavonoids and polyphenols in Radish Seed extract (Table 4). All of the aforementioned groups in the FT-IR analysis facilitate MB adsorption due to the formation of a physical bond.

**Table 4:** FT‒IR assignments of natural Raphanus seeds residual.

Wavenumber (cm^−1^)	Vibrational assignments
3415	‒OH stretching
2920	C–H stretching
2851	C–H stretching
2207	C≡C stretching
1615	C=O stretching
1112	‒CO stretching

### Adsorption of kinetics

#### Pseudo-first order model

The pseudo-first-order equation explains adsorption rate. Lagergren (1898) proposed a model of pseudo–first order kinetic which was given by Eq. (2):

4 $$\text{Ln }\left({\text{q}}_{\text{e}}-{\text{q}}_{\text{t}}\right)=\text{Ln}\left({\text{q}}_{\text{e}}\right)-{\text{k}}_{1}\text{t }$$

the form in Eq. (4) with boundary conditions of t = 0, q_t_ = 0 and t = t

5 $${\text{q}}_{\text{t}}=\frac{({\text{C}}_{0}-{\text{C}}_{\text{t}})\times \text{v}}{\text{m}}$$

6 $${\text{q}}_{\text{e}}=\frac{({\text{C}}_{0}-{\text{C}}_{\text{e}})\times \text{v}}{\text{m}}$$

**Figure 18:**
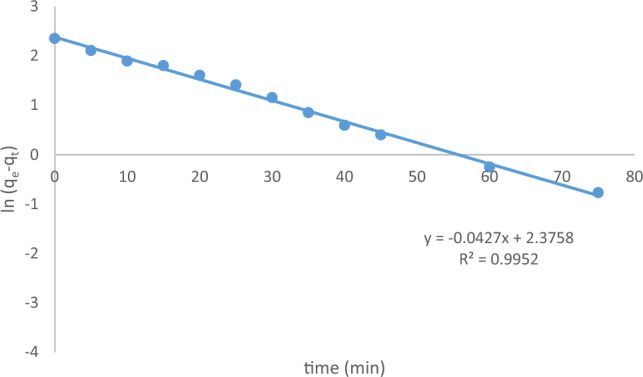
Pseudo-first order kinetic fit for MB plots for the adsorption onto Raphanus seeds solid residual (ACRS) at (initial concentration = 10 ppm, Raphanus seeds solid residual (ACRS) dose = 0.5 g/250 ml, stirring speed = 300 rpm, T = 301 K and time = 90 min, pH  7).

The plot of time (t) versus the natural logarithm of (q_e_ − q_t_) in Fig. 18 displays a linear relationship. The parameters q_e_ and k_1_ can be calculated from the intercept and slope of the graph, respectively^29^.

#### Pseudo-second order model

The pseudo-second order kinetic rate can be determined using the following Eq. (7):

7 $$\frac{{dq}_{t}}{dt}={k}_{2}{\left({q}_{e}-{q}_{t}\right)}^{2}$$

where k_2_ indicates the rate constant for the pseudo second order adsorption.(mg g^−1^ min^−1^). The Eq. (8) can be derived from Eq. (7) variables first be separated, then integrated, under the conditions (q_t_ = 0 at t = 0 and q_t_ = q_e_ at t = t) yielding a linear expression that describes the adsorption kinetics in a linearized integral form^20^:

8 $$\frac{\text{t}}{{\text{q}}_{\text{t}}}=\frac{1}{{\text{k}}_{2}{\text{q}}_{\text{e}}^{2}}+\frac{1}{{\text{q}}_{\text{e}}}\text{t}$$

**Figure 19:**
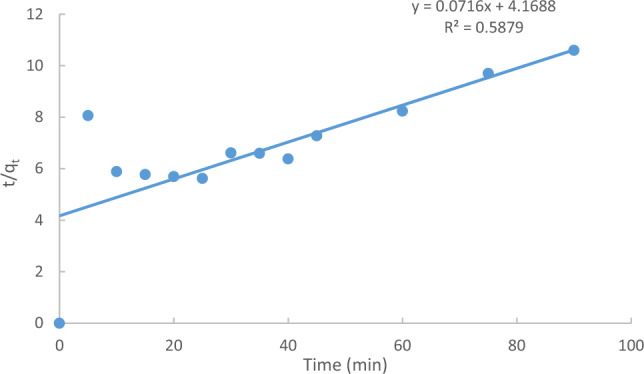
Pseudo-second order kinetic for the adsorption of MB onto Raphanus seeds solid residual (ACRS) at (initial conc. = 10 ppm, Raphanus seeds solid residual (ACRS) dose = 0.5 g/250 ml, stirring speed = 300 rpm, T = 301 K and time = 90 min, pH  7).

The integral form represented by Eq. (8) that indicated the ratio of time over the adsorbed amount of MB (t/q_t_) should be a linear function of time, as demonstrated in Fig. 19. Corresponding correlation coefficient values (R^2^) indicate that the pseudo-first-order model is better obeyed than the pseudo-second-order model. This is because the R^2^ value for the pseudo-first-order model is slightly higher than the R^2^ value for the pseudo-second-order model.

#### Weber and Morris model

The Weber-Morris model, which is also known as the model of intra-particle diffusion, is of significance in the field of liquid systems as it determines the rate of adsorption. Equation (9) provides a general representation of the kinetics involved in this model, where the intercept is a direct function of mass transfer across the boundary layer and the exponent is expected to have a value of 0.5

9 $${q}_{t}= {K}_{m}{t}^{0.5}+C$$

where: k_m_ is the intra- particle diffusion rate constant (mg/g min^1/2^).

**Figure 20:**
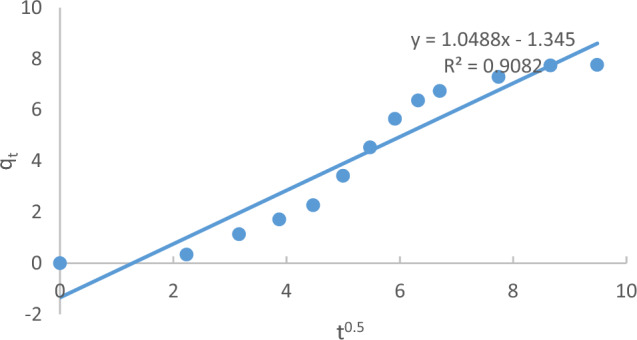
The Weber and Morris model for MB adsorption onto Raphanus seeds solid residual (ACRS) at (initial concentration = 10 ppm, Raphanus seeds solid residual (ACRS) dose = 0.5 g/250 ml, stirring speed = 300 rpm, T = 301 K and time = 90 min, pH  7).

The plot of qt versus t^1/2^ in Fig. 20 demonstrates a straight line with a slope (k_m_) and an intercept (C). The value of C represents an approximation of the boundary layer thickness, with a greater value indicating a thicker boundary layer.

In conclusion, the kinetic modeling of methylene blue (MB) adsorption onto Raphanus seeds solid residual (ACRS) was investigated using various kinetic models, namely the pseudo-first order, pseudo-second order, and Weber-Morris models.

For the pseudo-first-order model, Lagergren's kinetic equation was applied, revealing a linear relationship between the natural logarithm of (qe − qt) and time (t). The calculated parameters qe and k1 were obtained from the intercept and slope, respectively. However, this model may not be the most accurate representation, as indicated by the correlation coefficient (R^2^) values.

The pseudo-second-order model, described by the equation dq_t/dt = k2(qe − qt)^2^, was employed to assess the kinetic rate. The linearized integral form demonstrated that the pseudo-first-order model better adheres to the experimental data, with higher R^2^ values compared to the pseudo-second-order model.

Furthermore, the Weber-Morris model, focusing on intra-particle diffusion, was utilized. The linear plot of qt versus t1/2 revealed valuable insights into the mass transfer mechanism. The slope (km) and intercept (C) provided information about the intra-particle diffusion rate constant and boundary layer thickness, respectively.

Overall, the comparison of these kinetic models helps elucidate the dominant mechanisms governing the adsorption process. The pseudo-first-order model demonstrated a closer fit to the experimental data, suggesting its appropriateness for describing the kinetics of MB adsorption onto ACRS.

The adsorption of MB onto ACRS was studied under conditions of initial concentration of 10 ppm, contact time of 90 min, agitation rate of 300 rpm, and temperature of 25 °C. The results of the kinetics models and related parameters were calculated and presented in Table 5.

**Table 5:** Kinetic models parameters and the other parameters for adsorption of (MB) onto Raphanus seeds solid residual (ACRS) at initial concentration of (ACRS).

Kinetic models	Parameters	Initial concentration (10 ppm)
Pseudo first order equation	q_e_ (EXP.)(mg/g)	16.05
	q_e_ (calc.)	7.746
	R^2^	0.9952
	10^3^k_1_ (min^−1^)	68.1
Pseudo second order equation	q_e_ (EXP.) (mg/g)	16.05
	q_e_ (calc.)	7.746
	R^2^	0.0637
	10^3^k_2_ (g/mg min)	0.18
Weber and Morris model	C	−1.345
	R^2^	0.9082
	K_m_(mg g^−1^ min^−1^)	1.0488

### Thermodynamic parameters

The adsorption equilibrium data obtained at different temperatures were used to evaluate the important thermodynamic properties, including the standard Gibbs free energy (ΔG°), standard enthalpy change (ΔH°), and standard entropy change (ΔS°). The standard Gibbs free energy of the MB adsorption process was calculated using Eq. (8).^30^

10 $$\Delta {G}^{0}=-RTln{K}_{e}$$

The adsorption equilibrium constant (K_e_) can be determined for any temperature using Eq. (11).

11 $${K}_{e}=\frac{{q}_{e}}{{C}_{e}}$$

where C_e_ (mg/L) represents the equilibrium concentration of MB in the solution, R stands for the gas constant (8.314 J/mol·K), T denotes the absolute temperature in Kelvin, and q_e_ (mg/g) represents the amount of MB adsorbed from the solution at equilibrium.

12 $$\text{Ln }{\text{K}}_{\text{e}}= -\left(\frac{{\Delta \text{H}}^{0}}{\text{RT}}\right)+\left(\frac{{\Delta \text{S}}^{0}}{\text{R}}\right)$$

**Figure 21:**
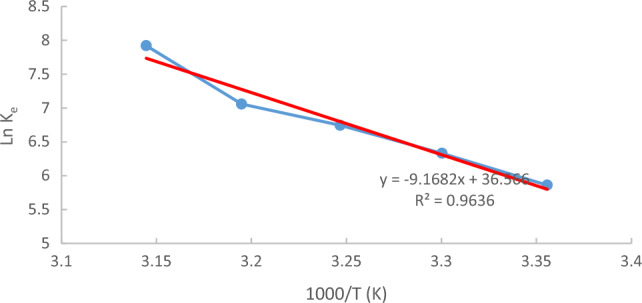
Van̕ t Hoff̓ s plot of the natural logarithm of the adsorption equilibrium constant at initial conc = 10 ppm, pH  7, Raphanus seeds solid residual (ACRS) dose = 0.5 g /250 ml and 300 rpm, contact time 90 min at different temperature.

Equation (12) displays ΔS° and ΔH°, which were obtained from the intercept and slope, respectively, of the Van't Hoff plot of 1/T versus ln(k_eq_) for MB, as depicted in Fig. 21.

Table 6 presents the values of ΔG°, ΔH°, and ΔS°, which illustrate the energetic properties of the divalent MB exchange. The positive ΔH° value suggests a high energy demand for the process, while the positive ΔS° value indicates a favorable interaction between the adsorbate and adsorbent. On the other hand, a negative ΔG° value implies that the adsorption process is feasible and spontaneous^31–34^^.^

**Table 6:** Thermodynamic characterization of MB adsorption at concentration 10 ppm, remove by (ACRS).

T (K)	298	303	308	313	318
1000/T (K^−1^)	3.355705	3.30033	3.246753	3.194888	3.144654
q_e_	7.76425	3.420033	2.773105	2.573326	3.049253
C_e_	0.022136	0.006087	0.003265	0.002214	0.001107
K_e_	350.75	561.8182	849.322	1162.5	2755
lnK_e_	5.860074	6.331178	6.744438	7.058328	7.921173
ΔG° (kJ/mol)	−14,518.8	−15,949.1	−17,270.6	−18,367.8	−20,942.4
∆H° (kJ/mol)	76.224				
ΔS° (kJ/mol K)	304.01				

### Isothermal models

Analysis of equilibrium data is a fundamental aspect in the evaluation of the maximum adsorption capacity of adsorbents. This analysis plays a crucial role in the determination of this capacity. Furthermore, it is critical to formulate an equation that accurately captures the experimental results, as this equation can then be utilized for design purposes. The Freundlich and Langmuir equations are the most commonly used models for the representation of adsorption equilibrium in an adsorption system^29,35^.

#### Langmuir adsorption isotherm

The assumption of homogeneous surface adsorption for the solute molecule MB was made, implying that the process occurs via monolayer adsorption without any interaction between the adsorbed species. The Langmuir equation is mathematically represented as Eq. (13)^30,36^:

13 $$\frac{{\text{C}}_{\text{e}}}{{\text{q}}_{\text{e}}}=\frac{1}{{\text{q}}_{\text{max}}\times \text{b}}+\frac{{\text{C}}_{\text{e}}}{{\text{q}}_{\text{max}}}$$

**Figure 22:**
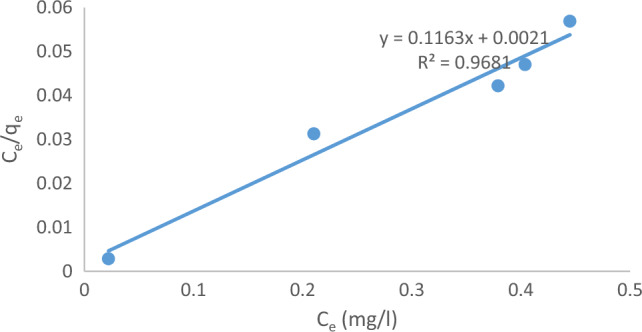
Langmuir adsorption isotherm for MB adsorption for solution of initial MB concentration = 10 ppm at pH  7, Raphanus seeds solid residual (ACRS) dose 0.5 g /250 ml at 300 rpm, contact time 90 min at temperature = 25 ℃.

In the context of adsorption, The term "q_e_" represents the equilibrium concentration of the adsorbate (methylene blue, MB) on the adsorbent, expressed in parts per million (ppm). "C_e_" represents the equilibrium concentration of MB in the solution, while "q_max_" represents the maximum achievable amount of MB that can adsorb onto the surface of the adsorbent, forming a monolayer. "b" is known as the Langmuir constant. The relationship between Ce and q_e_ can be visualized through a plot, which is shown in Fig. 22. This plot demonstrates that the adsorption of MB adheres to the Langmuir isotherm model, as evidenced by the linear relationship between Ce/q_e_ and Ce. The slope of this plot corresponds to the reciprocal of q_max_ (1/q_max_), while the intercept corresponds to the reciprocal of q_max_ times b (1/q_max_.b). The Langmuir isotherm can be characterized by a dimensionless constant referred to as the separation factor or equilibrium parameter, RL.

14 $${R}_{l}= \frac{1}{1+b\times {C}_{0}}$$

#### Freundlich adsorption isotherm

The Freundlich adsorption isotherm is a commonly employed mathematical model for fitting experimental data over a wide range of concentrations. This isotherm accounts for both surface heterogeneity and the exponential distribution of active sites and their energies. The Freundlich model is represented in a nonlinear fashion as follows^37^:

15 $${\text{q}}_{\text{e}}={\text{K}}_{\text{f}} {\left({\text{C}}_{\text{e}}\right)}^{1/n}$$

**Figure 23:**
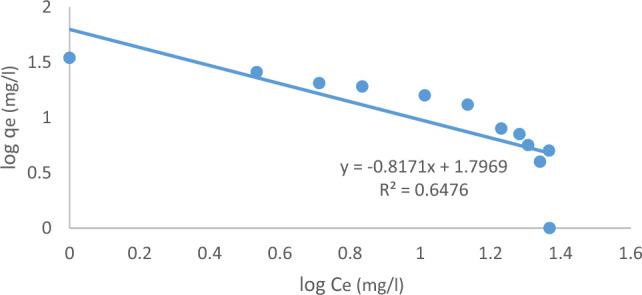
The Freundlich isotherm for MB adsorption was determined using an initial MB concentration of 10 ppm, at a pH of 7, with an ACRS dose of 0.5 g per 250 ml at a stirring speed of 300 rpm, a contact time of 90 min, and a temperature of 25 ℃.

The linear from of Freundlich model is expressed as follows:

16 $$\text{log}{q}_{e}=\text{log}{K}_{f}+\left(\frac{1}{n}\right)\text{log}{C}_{e}$$

where "K_f_" is the Freundlich constant representing the adsorption capacity and "n" is a constant related to the sorption intensity, which varies based on the heterogeneity of the adsorbent. A plot of log q_e_ versus log C_e_ results in a linear relationship with a slope (1/n) and an intercept (log K_f_), as depicted in Fig. 23^38,39^.

Table 7 provides a comparison between the Langmuir and Freundlich models. In the Langmuir model, the maximum monolayer sorption capacity q_max_ decreased with increasing doses of (ACRS), however in the Freundlich model.

**Table 7:** Langmuir isotherm constants and Freundlich isotherm constants in case of methylene blue.

Methylene blue	Langmuir isotherm constants			Freundlich isotherm constants		
	q_max_(mg/g)	b (L/mol)	R^2^	1/n	K_f_(mg/g)	R^2^
	8.6	55.381	0.9681	0.254	8.27	0.0832

## Conclusion

This study was conducted to examine the adsorption of methylene blue (MB) from an aqueous solution using natural activated carbon derived from Raphanus seeds solid residual (ACRS) as the adsorbent a novel good adsorbent used. The batch equilibrium method was utilized to analyze the adsorption of MB. The results showed that the adsorption capacity of ACRS is 99.4% and it was observed that the adsorption of MB was dependent on the initial concentration of the solution, the dosage of the adsorbent, and the initial pH of the solution. The relationship between the apparent surface area and weight of the sample was analyzed in relation to the amount of adsorbed pollutants, leading to the observation that natural activated carbon from Raphanus seeds solid residual (ACRS) exhibits a high adsorption capacity. The FT-IR analysis revealed that the MB adsorption was due to the formation of a physical bond between the MB and the ACRS. The results of the experiments were found to be in agreement with both the Langmuir and Freundlich isotherm models, with the Langmuir model being more favorable. The results of this study demonstrate that natural activated carbon derived from Raphanus seeds solid residual (ACRS) is a viable option for the removal of methylene blue (MB) from aqueous solutions. The optimization carried out using the Response Surface Methodology (RSM) confirms the conformity between the predicted and measured MB removal, indicating that RSM is a valuable method for optimizing and designing parameters in addition to its uses as an experimental design and statistical analysis tool. The application of RSM serves to streamline the experimental process by reducing the number of necessary trials, thereby minimizing the associated costs of extensive analyses. The Box–Behnken Design (BBD) was utilized within the RSM framework to optimize the percentage removal of methylene blue (%MB). The optimal conditions, as determined by RSM, include a pH of 7, a contact time of 120 min, an initial concentration of 10 ppm, ACRS dosage of 1 gm, and an adsorption temperature of 45 °C. These findings highlight the potential of ACRS under the specified conditions for efficient removal of cationic textile dyes from industrial wastewater.

### Permission to collect Raphanus seeds

There is no permission to collect Raphanus seeds because we not work on the seeds, we work on the residual of squeezing oils which is waste get rid of it. the plant seeds were bought from a shop called (Hagg Day for oil seeds). All procedures were conducted in accordance to the guidelines.

## Data availability

All data available in the paper and with the corresponding author.
